# The Position of Lingula as an Index for Inferior Alveolar Nerve Block Injection in 7-11-Year-Old Children

**DOI:** 10.5681/joddd.2010.013

**Published:** 2010-06-24

**Authors:** Fatemeh Ezoddini Ardakani, Zahra Bahrololoumi, Maryam Zangouie Booshehri, Alireza Navab Azam, Fatemeh Ayatollahi

**Affiliations:** ^1^ Associate professor of Oral and Maxillofacial Radiology Faculty of Dentisty, Shahid Sadough University of Medical Sciences Yazd, Iran; ^2^ Assistant professor of Pediatric Dentistry Faculty of Dentisty, Shahid Sadough University of Medical Sciences Yazd, Iran; ^3^ Assistant professor of Oral and Maxillofacial Radiology.Shahid Sadoughi University of Medical Sciences Yazd Iran; ^4^ Lecturer or Instructer of Oral and Maxillofacial Surgery,Shahid Sadoughi University of Medical Sciences Yazd, Iran; ^5^ Dentist. Faculty of Dentisty, Shahid Sadough University of Medical Sciences Yazd, Iran

**Keywords:** Block injection, inferior alveolar nerve, lingula, panoramic radiography

## Abstract

**Background and aims:**

Inferior alveolar nerve block injection is one of the common intra oral anesthetic techniques, with a failure rate of 15-20%. The aim of this study was to evaluate the position of the lingula as an index for this injection.

**Materials and methods:**

Thirty eight panoramic radiographs of 7–11 year old patients were analyzed and the distance between the lingula index and occlusal plane was measured. Then, lower alveolar nerve block injection was performed on 88 children. Finally, a visual analogue scale was used to measure the rate of pain in the patients.

**Results:**

This distance increased with age and in children younger than nine years is −0.45 mm on the right side and −0.95 mm on the left side. This distance in children older than 9 years is −0.23 mm on the right side and 0.47 mm on the left side. The success rates of inferior alveolar nerve block injection based on lingual index were 49% on the right side and 53.8% on the left side.

**Conclusion:**

As the lingual index has various positions and its distance from the occlusal plane increases with age, it is not an appropriate landmark for inferior alveolar nerve block injection.

## Introduction

Inferior alveolar nerve block or the mandibular nerve block is a common injection in dentistry. Unfortunately, the failure rate of this technique even with accurate injection is 15 to 20%. Absence of appropriate bony landmarks and big differences in dimensions of the ramus and position of the mandibular foramen are the reasons for failure of this technique.^1^ It can sometimes completely anesthetize the lower incisive primary teeth, but not the lower primary or permanent molars. The mandibular foramen in children is at a lower level than the occlusal level of the primary teeth and therefore the injection has to be administered lower and posterior to the position in adults.^1^

Lingula is a bony protrusion similar to the tongue that protects the mandibular foramen on the anterior part.^2^ In different populations, the lingula has different shapes like branching, nodular or triangular. The branching variety is more common in the adult Thai population. In addition to the position of the lingula, panoramic radiograph also shows the number of its branches.^3^ In more than 50% of adults, the lingula takes part in formation of half to two thirds of the wall of the mandibular foramen, and the myelohyoid line starts from the posterior border of the lingula.^4^

In order to evaluate the success rate of injection, the method of pain measurement has to be determined. The basic problem is that pain is a personal experience, which cannot be visualized or felt by another person, and indirect methods have to be used for this purpose. These methods include physiological, behavioral and report by the patient.^5^ The most common method is using a 10cm line with the extreme details at each end (no pain and extreme pain), which is called a visual analogue scale. The patients express pain by putting a cross on the line. However, this method can be affected by fear and expectation of pain by the patients.^5^ Despite this, observations show that visual analogue scale is one of the most reliable measurement tools for personal reporting of pain in children.^2^

Hannan et al^6^ compared the amount of desensitization gained by infra-alveolar block between the normal method and using ultrasound as a guide for the site of injection. A pulp tester was used to test the anterior and posterior mandibular teeth randomly in a 4-minute cycle for 60 minutes. The success rate was 38-92% in both methods and there was no significant difference in desensitization between the methods.^6^

Keros et al^7^ compared 50 panoramic radiographs related to successful infra-alveolar blocks with 94 other panoramic radiographs to non-successful infra-alveolar blocks. The results showed that the lingula was prominent in 56% of radiographs of those in whom the block was successful.^7^

The aim of the present study was to assess the use of the position of the lingula on the panoramic radiograph as a landmark for injection of infra-alveolar nerve block injection in 7-11-year-old children. For this purpose, the mean distance between the lingula and the occlusal plane was measured on panoramic radiographs and analyzed considering the age and gender variables. In a next stage, the success rate of the mandibular alveolar nerve block was assessed, using the mean distance of lingula and the occlusal plane as a landmark for needle insertion point.

## Materials and methods

**Figure 1:**
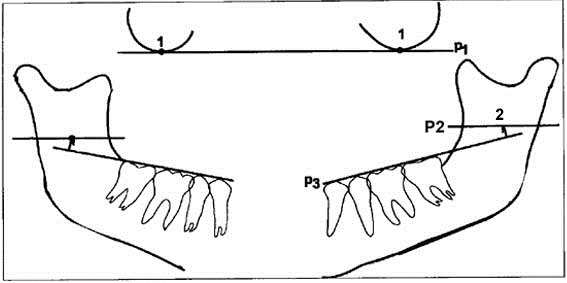
Measuring the ligula distance from the occlusal plane on the trace of a panoramic radiography.

This study was performed on 88 children, 7–11 years old, referring to the Faculty of Dentistry, Shahid Sadoughi University of Medical Sciences, Yazd, Iran, for filling or extraction of the second primary or first permanent molars. All subjects had a panoramic radiograph. Patients with a distorted panoramic radiograph and those who had a systemic disease like cardiovascular diseases, anemia or hemophilia were excluded from the study. All radiographs were taken at the Department of Oral and Maxillofacial Radiology, Shahid Sadoughi University of Medical Sciences, by one technician. Panoramic dental images were acquired with a Planmeca 2002 EC proline multitomographic X-ray unit (Planmeca Co., Helsinki, Finland),using a constant 12 mA, 80 KVP and 18 s exposure through 2.5 mm Aluminum filtration. Regular Kodak Lanex (Eastman Kodak Co, Rochester, NY) intensifying screens (15×30 cm cassette) and Kodak T Mat G films (Eastman Kodak Co, Rochester, NY) were used in this study. Films were developed in an automatic film processor (Velopex, Extra-X, Medivance Instruments Ltd, London, UK) with standard solutions. The total time of processing was 4 minutes at a working temperature of 27° C. A total of 38 panoramic radiographs with the necessary criteria were selected and assessed by a single examiner, an oral and maxillofacial radiologist with more than 10 years of experience. The technique for measuring the distance between the lingula and occlusal plane was as follows (Figure 1): Two orbital points were joined by one line (p1). Parallel with this line, two lines were drawn on the points of the lingula (p2). The occlusal plane was drawn on both side (p3) and the perpendicular distance between p2 and p3 was measured (Figure 1). The mean distance on both sides was calculated on the basis of age and gender and this data was used in the next stage.

1: Orbital point: the lowest point of the infraorbital margin

2: The most superior point of the mandibular lingula

P_1_: orbital plane: line to the orbital points

P_2_: plane parallel to the orbital plane passing through the most superior point of the mandibular lingula

P_3_: Occlusal plane: tangent to the tips of the cusps of the lower posterior teeth

In the 7–9-year-old age group, injection was administered at a distance of –0.45 mm on the right side and –0.95 mm on the left side in relation to the occlusal plane. While in the 9–11–year-old age group, injection was administered at a distance of –0.23 mm on the right side and 0.47 mm on the left side in relation to the occlusal plane. In all cases, 1.8 ml lidocaine 2% with 1:100000 epinephrine was injected using the direct technique and a 27 gauge long needle. Pain was measured by the visual analogue scale to determine the success rate of the injection. The technique was considered as successful when the child reported no pain and had selected the first 10% of the scale.

The data was analyzed using one-sample t-test, Chi-square and Mann-Whitney U-test. A confidence level of 95% was used for reporting and analyzing the results of the study.

## Results

There was no significant difference in the mean distance between the lingula and the occlusal plane on both sides in either sex (Table 1). In addition, there was no significant difference in the success rate of the inferior alveolar nerve block in the two age groups (Table 2).

**Table 1:** Distance between the lingula and occlusal plane on basis of gender (in mm)

Gender	n	Right side	SD	Left side	SD
Girls	23	- 0.565	2.29	- 0.435	2.73
Boys	15	- 0.033	2.39	- 0.133	3.92
Total	38	- 0.355	2.31	- 0.316	3.21
p-value			0.496	0.781	

(t-test)

**Table 2:** Success rate of inferior alveolar nerve block on basis of age

			Right side					Left side		
Age	n	Successful		Not successful			Successful		Not successful	
	Total	N	%	N	%	N	N	%	N	%
7-9	28	15	35.6	13	46.4	22	13	59.1	9	40.9
9-11	21	11	52.4	10	47.6	17	11	64.7	6	35.3
Total	49	26	53.1	23	46.9	39	24	61.5	15	38.5
p-value			0.934					0.721		

Chi-Square test: None of them are significant

Although there was no significant difference in the success rate of the inferior alveolar nerve block on the left side in both genders, but this rate was significantly different on the right side (Table 3).

**Table 3:** Success rate of inferior alveolar nerve block on basis of gender

			Right side					Left side		
Gender		Successful		Not successful			Successful		Not successful	
	Total	n	%	n	%	Total	n	%	n	%
Girls	29	18	62	11	37.9	21	12	57.1	9	42.9
Boys	20	8	40	12	60	18	12	66.7	6	33.3
p-value			0.128					0.542		

The injection on the right side was successful in 24/49 cases (49%). Of injections on the left side, 53.8% (21/39 cases) were successful. Chi-square test did not show any relationship between success rate and gender.

## Discussion

Inferior alveolar nerve block (mandibular nerve block) is one of the most common intra-oral anesthetic injections with challenging techniques. Malamed^8^ suggests that in order to perform the injection, the index finger or thumb should be placed on the coronoid fissure in order to obtain an imaginary line from the fissure to the pterygomandibular raphe that determines the height of the injection. This line is parallel and approximately 6–10 mm above the occlusal plane. In this technique, the dentist inserts the needle at the 2/3 or 3/4 anterior-posterior width of the ramus on this line. The horizontal landmark is an accurate and good reference, but the vertical line is not very specific.^8^

The present research studied the position of the lingula as a landmark for administration of infra alveolar nerve block. The injection was administered on the basis of the mean distance between the lingula and the occlusal plane in two age groups; 7-9 years and 9-11 years. The success rate of the technique was 53.8% on the left and 45% on the right side, which was lower than the overall success rate of inferior alveolar nerve block.

Quinn^8^ used the distance between mylohyoid ridge and supra lingual fissure as a reference for infra alveolar nerve block and concluded that mylohyoid ridge is the most specific reference for this injection, as it is nearer than the border of the ramus and external oblique ridge to the mandibular foramen, thus increasing the chances of the success rate. In the latter study, 2% lidocaine and 1:80000 epinephrine was used with the indirect method, whereas in the present study, 2% lidocaine and 1:100000 epinephrine was used with the direct method. These could be the reasons of the lesser success rate in the present study using the lingula as a reference in comparison to mylohyoid ridge as a reference for infra alveolar nerve block.

Hannan et al^6^ compared the rate of desensitization obtained after routine infra alveolar nerve block with that obtained after a block using ultrasound guide and found no significant difference between these two methods (p > 0.05). They concluded that the method of needle insertion is not the primary reason for failure of anesthesia in this technique.^6^ The results of the present study also show that the lingula is not an appropriate landmark for insertion of needle in an inferior alveolar nerve block.

Yonchak et al^9^ compared the rate of desensitization obtained after unilateral and bilateral mandibular nerve block. The success rate of the bilateral mandibular block was significantly higher than the unilateral mandibular block for central and lateral incisors. The chance of success rate for the desensitization of teeth in the bilateral mandibular block was 75% and therefore it was not recommended clinically.^9^ The results of the present study showed that the success rate of the infra alveolar nerve block using the lingula as a reference on both sides was lower than that of the latter study,^9^ but considering the fact that the failure rate in the routine method is also high, more studies are required to determine the appropriate position for insertion of the needle during an inferior alveolar nerve block.

Kanno et al,^10^ studying the position of the lingula as a reference for inferior alveolar nerve block injection, showed that the relative position of the lingula was constant. The results suggested that the infra alveolar injection should be administered 6 mm above the occlusal plane in 7-8-year-olds and 10 mm above the occlusal plane in 9-10-year-olds.^10^ The results of the present study show that the distance of the lingula from the occlusal plane increases with age and the success rate of the infra alveolar nerve block on basis of lingula as a landmark is lower than the normal routine method.^10^

In a study on 38 male cadavers with a mean age of 17 years old at the time of death, the distance between the lingula and the occlusal plane was measured 2.4 mm on the right, and 2 mm on the left side.^11^ The ideal point of insertion of the needle in inferior alveolar nerve block was proposed 8 mm above the occlusal plane.^11^ This more than the measurement of the present study. This could be due to a finding of the present study that the distance between the lingula and occlusal plane increases with age or the different methods of measurement used.^11^

The distance between the mandibular notch and mandibular foramen and the distance between the anterior ramus ridge and mandibular foramen were more than those in the group with successful nerve block, while the distance between the posterior ramus ridge and mandibular foramen and the distance between the mandibular angle and mandibular foramen was high in the group with an unsuccessful nerve block. The different locations of the lingula could be one of the reasons for failure of an infra alveolar nerve block.

## Conclusion

The distance between the lingula and the occlusal plane increases with age. The success rate of an inferior alveolar nerve block with lingula as a landmark is lower than the normal routine method.
